# Pyomyositis of the Biceps Brachii in a Young Adult Male

**DOI:** 10.7759/cureus.43582

**Published:** 2023-08-16

**Authors:** Dexter M Kirk, Taylor Brown, Christopher Yeary

**Affiliations:** 1 Research, Lincoln Memorial University DeBusk College of Osteopathic Medicine, Harrogate, USA; 2 Internal Medicine, Norton Community Hospital, Norton, USA; 3 General Surgery, Norton Community Hospital, Norton, USA

**Keywords:** intramuscular abscess, bicep abscess, infectious pyomyositis, bacterial pyomyositis, pyomyositis

## Abstract

Pyomyositis is a skeletal muscle infection mainly found in tropical regions. It commonly affects larger muscles, especially those of the hips. MRI tends to be the gold standard for diagnosis. *Staphylococcus aureus* remains the predominant causal organism in most cases of pyomyositis. Immunocompromised patients are more likely to be susceptible to this infection.

In our case, an immunocompetent 27-year-old male in rural southwest Virginia was found to have a large abscess in his upper arm. Contrast-enhanced CT scan was acquired prior to drainage, leading to the diagnosis of pyomyositis. Empiric treatment with IV vancomycin 1 g q 24 hours and piperacillin/tazobactam 3.375 mg q 8 hours, prompt incision and drainage, and negative pressure wound VAC led to a complete resolution of the infection.

## Introduction

Pyomyositis is a rare bacterial infection that expresses itself as an abscess wholly contained within a skeletal muscle [1]. Prevalence tends to be higher in tropical regions and occurs more often in children. The infection often presents itself in large muscles, most notably in the hip, and tends to be caused by *Staphylococcus aureus* [1]. The disease usually occurs in relation to other insults, such as injection drug use, local trauma, prior viral/parasitic myositis, and vigor exercise [2]. A stunted immune system from diseases such as diabetes mellitus, HIV/AIDS, cancers, and other autoimmune diseases tends to increase the risk of acquiring this infection [3]. Lack of diagnosis with MRI or CT and delayed treatment with IV antibiotics may lead to systemic septic shock [4]. The following case displays a rare occurrence of pyomyositis in an atypical patient population and site of infection.

## Case presentation

A 27-year-old male patient presented to the hospital with a three-week history of a large, erythematous, exquisitely painful abscess on the left upper arm. He stated he had broken his arm three to four months ago, and since the injury, he had noticed his arm swelling over the course of the next few months. The original fracture was noted to be an impacted fracture through the distal radius with a nondisplaced fracture of the ulnar styloid process. This fracture was noted to occur after a traumatic injury involving a quad all-terrain vehicle. He was placed in an immobilizer but did not follow up with orthopedic consultation. No further imaging other than an X-ray of the wrist was performed at that time. The patient attempted conservative management for the swelling of the left bicep at home. Still, the pain and size of the abscess continued to escalate, leading to his presentation to the emergency department. He denies subjective fever, insect bites, intravenous drug use, prior evaluation, or recent sick contacts or travel. The additional past medical history was negative. The patient had a previous surgical history of septoplasty. He endorsed cigarette use of 1.5 packs per day and vaping of synthetic marijuana. Abnormal laboratory data were significant for leukocytosis only (20,100 WBC/mm).

**Figure 1:**
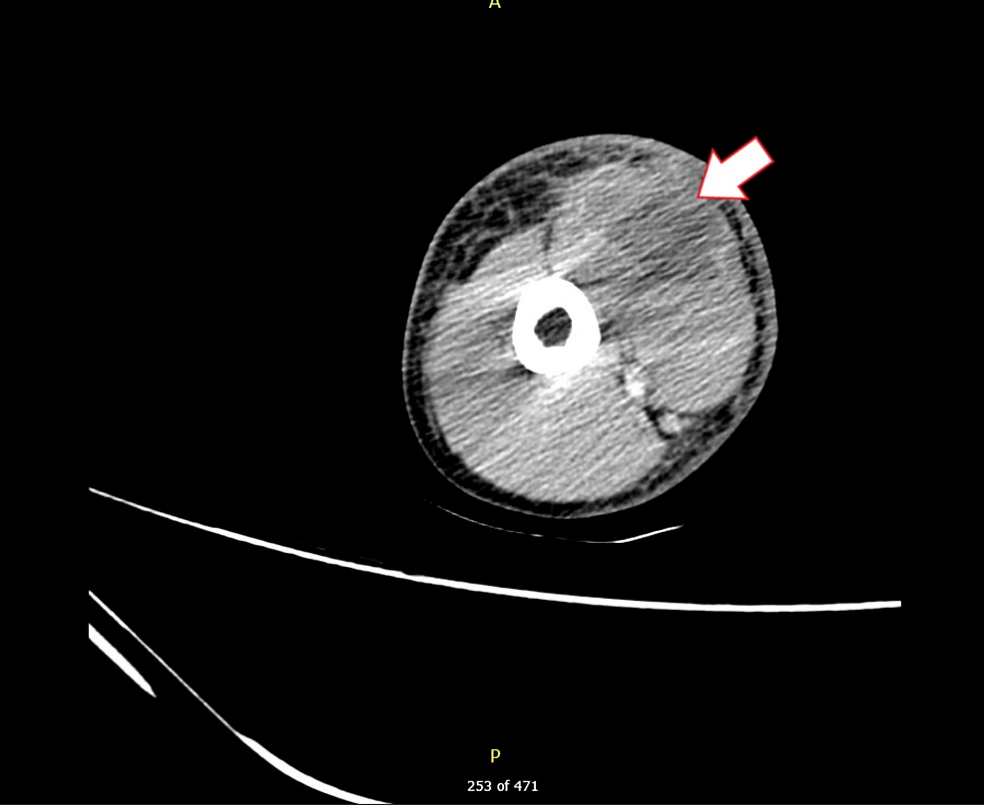
Computed tomography with intravenous contrast of the left upper extremity displaying a 2.3 × 3.4 × 4.8 cm collection contained within the biceps brachii

Computed tomography of the left humerus with IV contrast was performed. No osseous abnormality of the left humerus nor joint effusion was found. A subtle peripherally enhancing fluid collection was seen in the anterior left brachium, which involved the biceps brachii muscle and was measured at 2.3 × 3.4 × 4.8 cm. Subcutaneous edema was also appreciated along the anterior brachium from the shoulder to the elbow without lymphadenopathy. The radiographic impression was an abscess consistent with pyomyositis, with soft tissue edema consistent with cellulitis (Figure 1). 

The patient was given IV vancomycin and piperacillin/tazobactam. General surgery was then consulted. During the examination, the pain was exacerbated by both passive and active movement. It was warm to the touch and tender upon palpation. No crepitus was appreciated; however, an apparent fluctuant abscess was present. All compartments of the left upper extremity were soft, and ulnar/radial pulses were appreciated on the ipsilateral side. The patient was noted to have a slightly decreased range of motion on the elbow flexion. The patient was then taken to the operating room for surgical incision and drainage.

After the incision, the abscess drained copious amounts of malodorous, purulent, and bloody fluid. Cultures were obtained, and the wound was promptly drained and irrigated with 1.5 L of saline. The biceps brachii muscle was noted to have a deep abscess cavity posteriorly along the short head of the biceps brachii. There were no signs of necrosis or extension of abscess along the fascial planes. Negative pressure wound dressing was applied. Intraoperative wound cultures returned as *Streptococcus anginosus*. Gram stain returned as moderate white blood cells with no organisms seen. After incision and drainage, the patient was sent home with oral amoxicillin-clavulanate 875/125 mg BID for additional seven days.

On the two-week follow-up, the patient stated that the pain and tenderness had subsided. The patient was noted to have a full range of motion of the bicep. The wound was well-healed, and the negative pressure wound device was removed. The wound itself was noted to be flush with the skin with good granulation tissue. The patient was released from care and has had no recurrence within the past year.

## Discussion

Pyomyositis has mainly been documented as most likely occurring in children and tropical regions and commonly infecting large hip muscles [1]. These demographics may tend to raise suspicion of this infection as a probable diagnosis. As in our case, the individual did not fall within these demographics, leading to the assumption of a subcutaneous abscess prior to imaging. Thorough examination and imaging must be conducted to promptly treat before any worse complications may arise. 

Though commonly thought to be relegated to hip musculature, Ashken and Cotton described the infection as occurring specifically in large skeletal muscles, such as the serratus anterior, pectoralis major, biceps, quadriceps, glutei, gastrocnemius, iliopsoas, and abdominal and spinal muscles [5]. Of note, very little literature mentions the infection found in biceps brachii. Tatsuno et al. described a case in which an older male presented with right shoulder pain was diagnosed with pyomyositis in both the bicep brachii and pectoralis major [6]. 

In a meta-analysis performed in 2021 by Ngor et al., it was found that one of the most significantly associated risk factors was HIV infection, in which individuals who are infected would be five times more likely to be diagnosed with pyomyositis than those who are uninfected [3]. Ngor et al. also found that a majority of patients diagnosed with pyomyositis were males fewer than 20 years old, with up to 90% of cases identifying *S. aureus* as the principal organism [3]. It is mentioned within Ngor et al.’s report that local trauma may be a predisposing factor to the development of pyomyositis [3]. Our case lies in contrast to this, with the patient being older than the most common cases and *S. anginosus* being cultured from the wound. A PubMed search of “pyomyositis” and “streptococcus anginosus” yielded five total cases involving this bacteria. 

Diagnosis using CT with contrast has shown to be effective, though contrast-enhanced MRI is still the gold standard [3,7]. These imaging modalities tend to only be sensitive in the later stage of infection, during which an abscess forms. The diagnosis of pyomyositis in our case was acceptable with an IV contrast CT prior to treatment. This swift diagnosis prevented a prolonged wait for treatment of the already progressed condition. 

This case is particularly interesting, as an adult male in rural Virginia was found to have pyomyositis with progression to a purulent abscess contained within his bicep. Given the paucity of inoculation of the soft tissues or immunocompromised status, this case is of interest to the literature. With his traumatic history several months prior, it can be speculated that pyomyositis etiology could have arisen as a complication of muscular hematoma that was undiagnosed at that time. Without imaging, the abscess on the physical exam appeared to be strictly subcutaneous. Prompt IV antibiotic treatment followed by incision and drainage was significant to prevent the loss of muscle of the bicep and the progression of the disease to osteomyelitis, compartment syndrome, muscular damage, or septic shock. Swift imaging and including pyomyositis as a differential diagnosis are considered important to prevent these complications.

## Conclusions

Pyomyositis is a rare infection of skeletal muscle that may present similarly to a cutaneous abscess. The suspected abscesses require a thorough history, physical examination, and advanced imaging in order to direct treatment and prevent further complications.

## References

[1] Bickels J, Ben-Sira L, Kessler A, Wientroub S (2002). Primary pyomyositis. J Bone Joint Surg Am.

[2] Chattopadhyay B, Mukhopadhyay M, Chatterjee A, Biswas PK, Chatterjee N, Debnath NB (2013). Tropical pyomyositis. N Am J Med Sci.

[3] Ngor C, Hall L, Dean JA, Gilks CF (2021). Factors associated with pyomyositis: a systematic review and meta-analysis. Trop Med Int Health.

[4] Hiddema J, Hassan S, Mangat N, Siddiqui N (2017). Pyomyositis of the pectineus muscle in an adolescent male. Ann R Coll Surg Engl.

[5] AS MH, CO RE (1963). Tropical skeletal muscle abscesses (pyomyositis tropicans). Br J Surg.

[6] Tatsuno S, Reed T, Tatsuno E, Lee C (2020). 63-year-old man with right biceps and right pectoralis major abscesses: an unusual case of pyomyositis. BMJ Case Rep.

[7] Bharathi RS, Naveen S, Seth N (2011). Primary obturator internus pyomyositis. Med J Armed Forces India.

